# A long-term surviving patient with recurrent low-grade serous ovarian carcinoma treated with the MEK1/2 inhibitor, selumetinib

**DOI:** 10.1186/s40661-016-0026-5

**Published:** 2016-05-05

**Authors:** Munetaka Takekuma, Kwong K. Wong, Robert L. Coleman

**Affiliations:** The Division of Gynecology, Shizuoka Cancer Center, Shizuoka, 411-8777 Japan; Department of Gynecologic Oncology & Reproductive Medicine, University of Texas, M.D. Anderson Cancer Center, 1155 Herman Pressler Dr., CPB6.3590, Houston, TX 77030 USA

**Keywords:** Low-grade serous ovarian cancer, MEK inhibitor, Selumetinib, *KRAS* mutation

## Abstract

**Background:**

Selumetinib is a potent, selective, orally available, and non-ATP competitive small molecule inhibitor of mitogen-activated protein kinase kinase 1/2 (MEK1/2) that has demonstrated single agent activity in a number of solid tumor including recurrent low-grade serous ovarian carcinoma (LGSOC). However, the long-term prognosis of patients who receive selumetinib, as well as the late toxicity of the agent, have not yet been described.

**Case Presentation:**

In this case report, we present a patient with recurrent LGSOC with KRAS mutation whose tumor has not progressed and who has maintained a good general condition without severe toxicities following treatment with selumetinib for more than 7 years. Next generation sequencing of her tumor revealed a G12V mutation in KRAS. MAPK signaling inhibition plays a role in the biology of LGSOC.

**Conclusions:**

Although biomarkers have yet to definitively define patients with LGSOC who are likely to respond to therapy, exploration of specific alterations should be pursued in an excersie to develop a reliable companion diagnostic test.

## Background

Serous carcinoma is the most common histological type of ovarian cancer accounting for 70–80 % of all new diagnoses. Evaluation of the molecular biology of high-grade serous ovarian cancer (HGSOC) describes a disease associated with frequent P53 alterations, including hemizygous loss, mutation, or amplification. The natural history of HGSOC has been well documented and despite significant improvements in surgical and adjuvant treatment options, the number of patients cured has altered very little over the last two decades. International Federation of Gynecology and Obstetrics (FIGO) grading (grades 1, 2, and 3) has also been a well-documented prognostic factor for many of its histological subtypes. However, investigation in the early 1990’s suggested that low-grade serous ovarian cancer (LGSOC) could be most reliably identified through a bivariate classification (low and high). Further, patients with LGSOC appeared to have a much different natural history. [1] For instance, several studies have documented that LGSOC is not as sensitive to cytotoxic chemotherapy relative to its HGSOC counterpart but it is associated with much longer expected overall survival [2, 3]. In addition, molecular and genomic studies have shown that LGSOC appears to have a higher frequency of functional estrogen (ER) and progesterone receptor (PR) expression, *KRAS* or *BRAF* mutations, a higher frequency of expression of active mitogen-activated protein kinase (MAPK), and a lower frequency of *TP53* mutations than does HGSOC [4–7].

Selumetinib is a potent, selective, orally available, and non-ATP competitive small molecule inhibitor of mitogen-activated protein kinase kinase 1/2 (MEK1/2) that has demonstrated single agent activity (objective responses) in a number of solid tumor including recurrent LGSOC [8, 9]. Herein, we present the clinical experience of a woman with chemo- and hormonally-refractory recurrent LGSOC associated with a *KRAS* mutation whose tumor has continually responded to selumetinib for more than 7 years.

## Case presentation

A 40-year-old woman with bilateral ovarian masses was found at laparotomy to have a suspected ovarian malignancy in October 1997. Surgical extirpation included supracervical hysterectomy, bilateral salpingo-oophorectomy, appendectomy, and staging biopsies. The pathology was confirmed as a low-grade papillary serous carcinoma of the ovary arising from a tumor of low malignant potential in both ovaries with microscopic metastatic implants in the omentum and peritoneal surfaces. She was staged as FIGO Stage IIIA and was treated with post-operative intravenous paclitaxel (175 mg/m^2^) and carboplatin (AUC 5, PC) for six cycles.

She did well until October 2005, when the patient’s serum cancer antigen 125 (CA125) level was found to be increased and imaging studies showed recurrent peritoneal tumor. Biopsy confirmed recurrent LGSOC and she underwent a secondary cytoreduction. Final pathology also revealed ER and PR expression. Unfortunately, the distribution of her recurrent disease precluded complete surgical resection, leaving measurable disease in the small bowel mesentery. She was treated adjuvantly with nine cycles of PC achieving stable disease as her best response. Due to neuropathy, fatigue and marrow exhaustion she was not able to undergo further PC treatment. She was then treated with letrozole, an aromatase inhibitor, at a dose of 2.5 mg daily for 19 months. In May 2008, the patient’s imaging studies showed unequivocal progression of a left upper quadrant lesion, and the letrozole was discontinued.

**Fig. 1:**
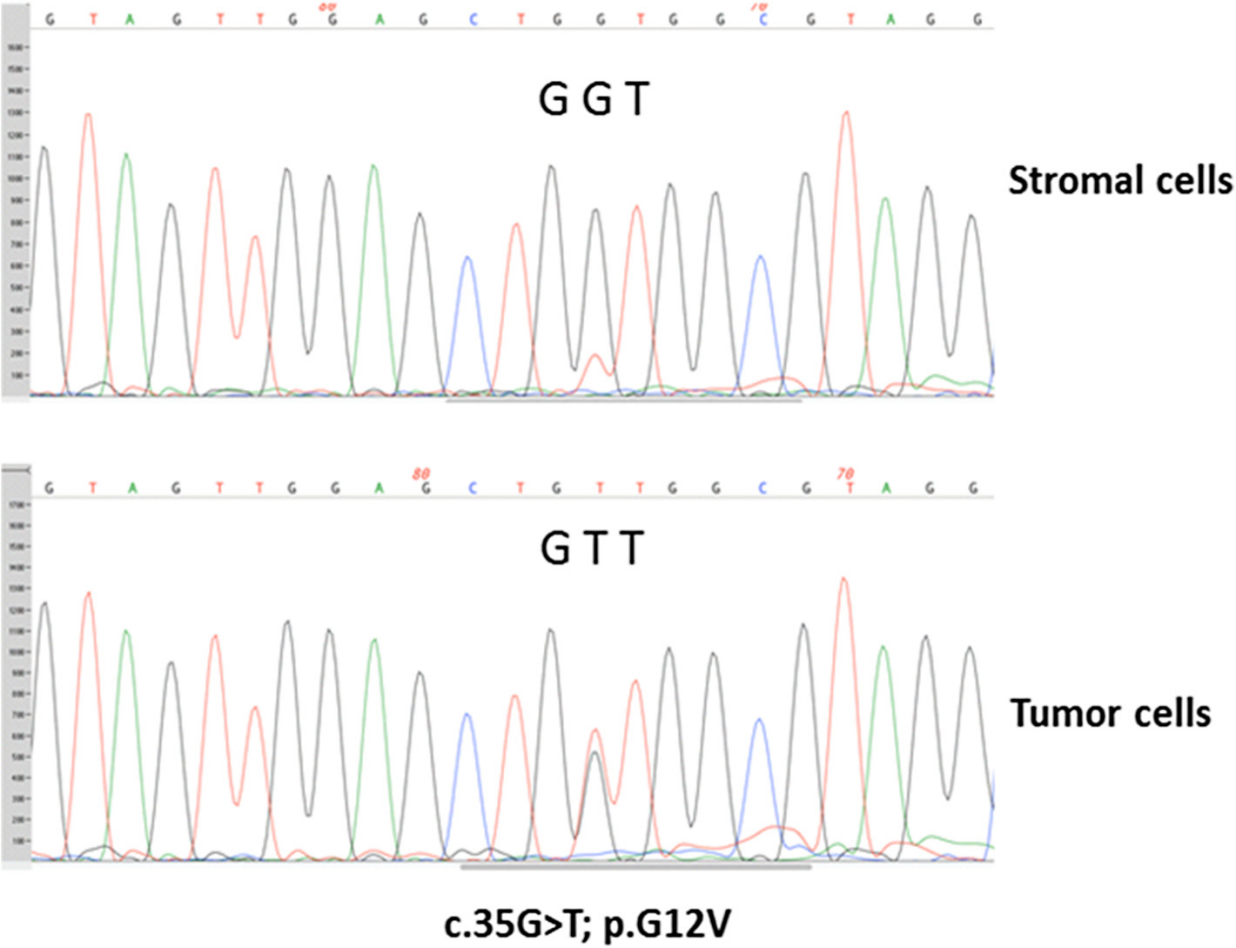
Detection of a somatic KRAS mutation (c.35G > T; p.G12V) in DNA extracted from micro-dissected tumor cells from the patient with recurrent low-grade serous carcinoma. DNA extracted from micro-dissected adjacent stromal cells was used as normal control. Sequencing chromatograms were generated using PCR Sanger sequencing with AB3730XL sequencer

**Fig. 2:**
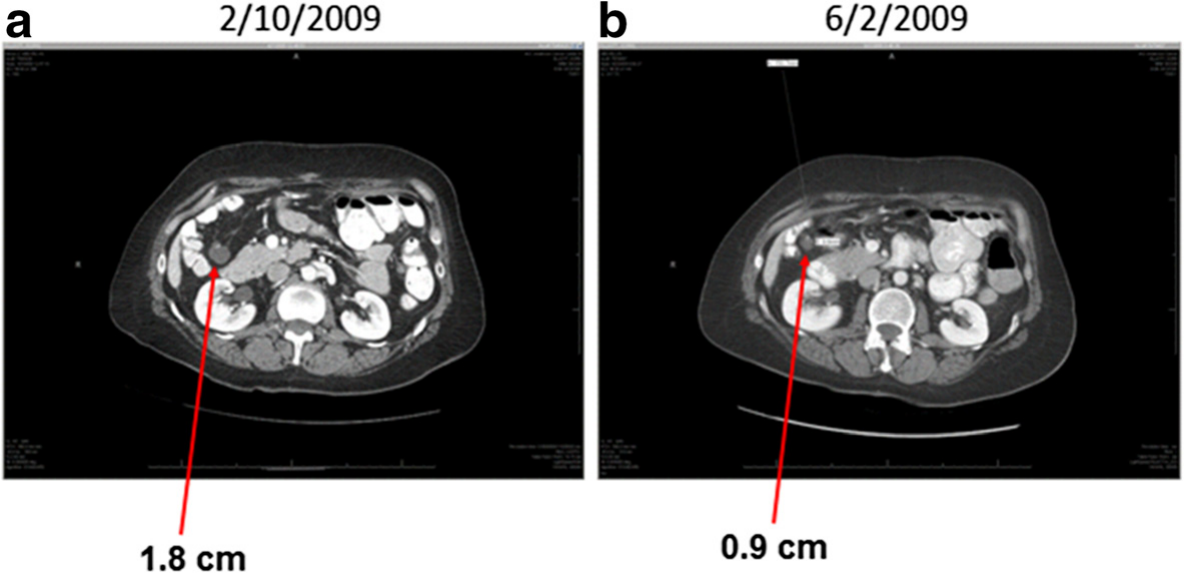
Computerized tomography (CT) scans showing a decrease in an implant close to the hepatic flexure of the colon after the patients have started taking selumetinib since June 2008

In consideration of additional therapeutic options and clinical trials, we performed a mutational analysis of the recurrent tumor by next generation sequencing (NGS) [10]. This revealed a *KRAS* mutation (Fig. 1). Fortunately, GOG-239, a phase II clinical trial of selumetinib in patients with LGSOC became available at this time, to which she enrolled in June 2008. Selumetinib was administered orally at a dose of 50 mg twice daily. A computerized tomography (CT) scan was performed in February 2009, and no new implants, ascites, masses or adenopathy were seen in the abdomen or pelvis. The impression was that there were stable implants. An implant close to the hepatic flexure of the colon had a measurement of 1.8 cm (Fig. 2a). Subsequent CT imaging scan done in June 2009 showed sufficient change in her measurable disease, including the previous hepatic flexure implant (0.9 cm, Fig. 2b) to register a partial response. She has continued on this therapy nearly uninterrupted since. Most of her multiple recurrent lesions, which were frequently associated with calcific deposits have lost their soft tissue component and/or cystic nature. Concern for lack of active disease, she has subsequently undergone several PET-CT images reveal FDG-avid implants. In addition, tumor growth was observed following two treatment breaks required for care of other medical issues unrelated to disease or therapy. Remarkably, reinitiation of selumetinib resulted in tumor implant size reduction and for one lesion, resolution.

Our patient has experienced mild adverse events over the many years of therapy. Most notable were intermittent rashes (maximum grade 2) at anatomically disparate areas, such as the trunk, chest, and eyelids, each of which were treated conservatively with antibiotic ointments and topical antihistamines. She has also experienced grade 1 stomatits, intermittently. Of most notable effect, she experienced de-pigmentation of her hair (now blonde) and a birthmark on her right arm (no longer visible). During the duration of her treatment the formulation of selumetinib administered was changed from a daily sachet (“mix and drink”, 100 mg BID) to tablets (50 mg BID). Initially, this patient’s adverse events did increase in frequency and intensity during the transition, although they did not require discontinuation of the therapy and further administration was not compromised. Continuous monitoring for MEK-related cardiovascular, ophthalmological and metabolic dysfunctions are ongoing.

## Discussion

We present an unusual patient with recurrent LGSOC with a *KRAS* mutation (G12V) whose tumor has not progressed and who has maintained a good general condition without severe toxicities by means of treatment with selumetinib, a MEK inhibitor, for more than 7 years. We believe she is the longest continuously treated patient with MEK-therapy under any indication.

As mentioned, this patient continues to participate in GOG-239, a phase II trial of selumetinib in women with recurrent, measurable LGSOC. Farley et al. recently reported the results from this trial, and demonstrated an overall response rate was 15 %, median progression-free survival was 11 months (interquartile range [IQR] 3.6–15.9) [9], and the median follow-up time in patients who survived was 21 months (IQR 17–30). At the time of the publication (reflecting information gathered until database lock), nine patients (17 %) had been on therapy for more than 15 months.

LGSOCs typically have an indolent course with recurrences developing over many years, but eventually these tumors develop intra-abdominal carcinomatosis, which proves to be fatal, and LGSOC has been recognized as more chemoresistant than HGSOC [2, 3]. In 2008, Schmeler et al. reported their experience with 25 patients with LGSOC treated with neoadjuvant chemotherapy for inoperable disease at diagnosis; all patients were treated with a platinum-based regimen, and only one (4 %) experienced a complete response, while none had a partial response, 21 had stable disease, and two (8 %) had progressive disease [2]. In 2009, Gershenson et al. published an analysis of 58 patients with recurrent LGSOC in which the overall response rate was 3.7 %; dividing the regimens by platinum-status, the response rate was 4.9 % in platinum-sensitive tumors and 2.1 % in platinum-resistant tumors, but that difference did not reach statistical significance (*p* = 0.63) [3]. Since the response rate of LGSOC to conventional treatment is extremely poor, investigation into new therapeutic approach is urgently needed.

The clinical efficacy of hormonal therapy for LGSOC is modest. A 2012 report from the M.D. Anderson Cancer Center documented an overall response rate and stable disease rate of 9 and 62 %, respectively [11]. There was a statistically significant relationship between response and platinum-status in terms of both overall response rate (ORR, 13.5 % vs 2.7 %) and time to progression (TPP, 8.9 vs 5.7 months, *P* = 0.003).

The mitogen-activated protein kinase (MAPK), also known as extracellular signal-regulated protein kinase (ERK), is a downstream target of RAS, RAF, and MAP/ERK kinase and is crucial for transduction of growth signals from several key growth factors, cytokines, and proto-oncogenes [12]. Mutations or overexpression of components, including *KRAS* and *BRAF,* in the MAPK pathway leads to constitutive activation of MAPK by phosphorylation. Activation of MAPK in turn activates downstream protein kinases, nuclear proteins, and transcription factors [13], which may contribute to the development of various tumors [14].

MEK is an attractive therapeutic target, as it lies downstream of multiple activators of the pathway. As such, selumetinib would be considered to have its greatest effects on tumors harboring *RAS* or *BRAF* activating mutations. While activating *RAS* mutations have been documented as a frequent event in serous borderline tumors and LGSOC, [14–16] there has been poor correlation between response to selumetinib and *RAS* or *BRAF* mutation. Of interest, G12D and G12V are the two most common *KRAS* mutations. However, their downstream signaling is not completely the same. G12D variant signals primarily through PI3K, JNK, p38, and FAK signaling pathways [17, 18] but signals less through the RAF/ERK pathway. Furthermore, G12D causes constitutive PI3K/mTOR activity [18]. One the other hand, the G12V variant signals predominantly through MAPK signaling cascade but has lost the ability to bind to and signal through PI3K [18]. Thus, we speculate that tumor cells with G12V mutation will be more sensitive to MEK inhibitor while those with G12D will require inhibitors targeting both MEK and PI3K pathways. Alternatively, additional mutation(s) in the tumor cells may contribute to the sensitivity to selumetinib. Using low passage (10X) whole genome sequencing, we have identified 9 additional potential somatic missense mutations (Table 1). Most of the amino acid changes have no predictive functional impact on the protein function except *USP45* gene. USP45 encodes an ubiquitin-specific protease, which may modulate the MAPK pathway [19]. Thus, further investigation is warranted. In addition, despite intensive studies, the genetic and molecular basis for resistance to selumetinib remains poorly understood. Grasso et al. reported that p70 S6 kinase and its downstream target ribosomal protein S6 may be biomarkers of resistance to selumetinib in colorectal cancer [20].

**Table 1:** Low 185 passage whole genome sequencing

		Tumor DNA			Blood DNA			
Gene	Amino acid change	Mutant allele reads	Wild-type allele reads	Frequency of mutant allele	Mutant allele reads	Wild-type allele reads	Frequency of mutant allele	Functional impact
CCDC110	P209Q	7	9	44	0	8	0	Neutral
FAT1	Q2933P	6	6	50	0	6	0	Neutral
PCDHGA1	A443T	7	6	54	0	5	0	Neutral
USP45	K67E	6	6	50	0	5	0	Medium
RP1L1	Q1861P	5	7	42	0	9	0	Neutral
PLBD1	L25delL	7	7	50	0	9	0	Neutral
KRAS	G12V	8	11	42	0	9	0	Medium
KRT84	I206V	11	4	73	0	3	0	Neutral
ADAMTS17	N1094S	6	7	46	0	8	0	Neutral
ADAM33	M764T	7	9	44	0	4	0	Low

## Conclusions

Treatment of LGSOC continues to evolve as new targets emerge with functional consequence. Our case report and others point to MAPK signaling as one pathway with therapeutic viability. Currently, two prospective, randomized phase III clinical trials of single agent MEK inhibitors (binimetinib and trametinib, both versus physician’s choice chemotherapy or hormones) are being conducted in women with LGSOC (NCT01849874, NCT02101788). Both trials require central pathology review to confirm the diagnosis and allow MEK inhibitor cross-over on progression of control therapy. Although recruitment to the former, using binimetinib, was recently halted due to an unfavorable planned futility assessment, a comprehensive review of the final results from these trials will be important to determine the role MEK inhibitors in this rare disease.

### Patient consent

Written informed consent was obtained from the patient for publication of this Case Report and any accompanying images. A copy of the written consent is available for review by the Editor-in-Chief of this journal.
